# Biocompatibility and Mineralization Activity of Three Calcium Silicate-Based Root Canal Sealers Compared to Conventional Resin-Based Sealer in Human Dental Pulp Stem Cells

**DOI:** 10.3390/ma12152482

**Published:** 2019-08-05

**Authors:** Deog-Gyu Seo, Donghee Lee, Yong-Min Kim, Dani Song, Sin-Young Kim

**Affiliations:** 1 Department of Conservative Dentistry, Dentistry and Dental Research Institute, School of Dentistry, Seoul National University, Seoul 03080, Korea; 2 College of Medicine, The Catholic University of Korea, Seoul 06591, Korea; 3 Department of Conservative Dentistry, Seoul St. Mary’s Dental Hospital, College of Medicine, The Catholic University of Korea, Seoul 06591, Korea

**Keywords:** cell viability, cell migration, scanning electron microscope, mineralization, calcium silicate-based sealer, conventional resin-based sealer

## Abstract

The purpose of this study was to compare the cytotoxic effects and mineralization activity of three calcium silicate-based root canal sealers to those of a conventional resin-based sealer. Experiments were performed using human dental pulp stem cells grown in a monolayer culture. The root canal sealers tested in this study were EndoSequence BC Sealer (Brasseler), BioRoot RCS (Septodont), Endoseal MTA (Maruchi), and AH Plus (Dentsply DeTrey). Experimental disks 6 mm in diameter and 3 mm in height were made and stored in a 100% humidity chamber at 37 °C for 72 h to achieve setting. The cytotoxicity of various root canal sealers was evaluated using a methyl-thiazoldiphenyl-tetrazolium (MTT) assay. To evaluate cell migration ability, a scratch wound healing method was used, and images of the scratch area were taken using a phase-contrast microscope. Cell morphology was evaluated by a scanning electron microscope after direct exposure for 72 h to each sealer disk. In the cell viability assay, there were no significant differences between the EndoSequence BC, BioRoot RCS, Endoseal MTA, and control groups in any experimental period (*p* > 0.05). In the cell migration assay, there were no significant differences between the EndoSequence BC, Endoseal MTA, and control groups in any experimental period (*p* > 0.05). BioRoot RCS exhibited slower cell migration relative to EndoSequence BC and Endoseal MTA for up to 72 h (*p* < 0.05). Conversely, it showed a similar wound healing percentage at 96 h (*p* > 0.05). In an evaluation of cell morphology, cells in direct contact with EndoSequence BC, BioRoot RCS, and Endoseal MTA disks showed superior spreading compared to those in contact with the AH Plus disk. In an Alizarin red staining assay, EndoSequence BC, BioRoot RCS, and Endoseal MTA showed a significant increase in mineralized nodule formation compared to the AH Plus group (*p* < 0.05). In conclusion, all calcium silicate-based root canal sealers tested in this study showed good biological properties and mineralization activity compared to conventional resin-based sealer.

## 1. Introduction

The aim of root canal treatment is to provide three-dimensional obturation of the root canal system to prevent the entry of bacteria and fluid [1,2,3]. To provide hermetic sealing, core materials such as gutta-percha (GP) and root canal sealers are essential [4,5]. Sealers should be biocompatible and nonirritating to the periradicular tissues [6,7]. The most commonly used sealers are calcium hydroxide sealers, glass ionomer sealers, zinc oxide eugenol sealers, resin-based sealers, and the recently introduced calcium silicate-based sealers. AH Plus Plus (Dentsply DeTrey GmbH, Konstanz, Germany) is a conventional epoxy resin-based root canal sealer with low microleakage and good penetration ability into dentinal walls [8,9]. This sealer exhibits toxicity when freshly mixed that gradually reduces upon setting [7,10]. If in direct contact with viable tissues over extended periods, it causes an inflammatory reaction and may result in delayed healing [11]. To overcome this toxicity problem, calcium silicate-based sealers have been developed.

EndoSequence BC Sealer (Brasseler, Savannah, GA, USA) is a bioceramic calcium phosphate silicate-based sealer. It has an alkaline pH, a high rate of calcium ion release, and suitable flow capacity [12]. It also shows superior biocompatibility and osteogenic differentiation ability relative to other endodontic sealers [13,14,15,16,17]. 

BioRoot RCS (Septodont, Saint Maur-des-Fossés, France) is composed mainly of tricalcium silicate and zirconium oxide powder that must be mixed with a liquid containing calcium chloride. In recent studies comparing epoxy resin-based and calcium silicate-based sealers, BioRoot RCS showed excellent biocompatibility in both fresh and set states [17,18,19]. 

Endoseal MTA (Maruchi, Wonju, Korea) is a pozzolan-based premixed calcium silicate sealer that offers satisfactory biocompatibility [15,20] and good root canal filling quality [21,22]. It has high alkalinity and low solubility, similarly to ProRoot MTA (Dentsply Tulsa Dental Specialties, Johnson City, TN, USA) [23].

The biocompatibility and osteogenic potential of these three newly developed root canal sealers have not been fully reported. The purpose of this study was to compare the cytotoxic effects and mineralization activity of the above three calcium silicate-based root canal sealers to those of a conventional resin-based sealer. 

## 2. Materials and Methods

### 2.1. Human Dental Pulp Stem Cells (hDPSCs)

This study was approved by the institutional review board of the Catholic University of Korea (IRB No. KC19SNSI0186). Passage 3 hDPSCs were used for the experiments in this study. The cell lines were developed anonymously at Top Cell Bio, Inc. (Seoul, Korea). Cells were grown in a growth medium consisting of HyClone Minimum Essential Medium (α-MEM; GE Healthcare Life Sciences, Pittsburgh, PA, USA) supplemented with HyClone 10% fetal bovine serum (GE Healthcare Life Sciences), 100 U/mL of penicillin, and 100 μg/mL of streptomycin. Cell cultures were maintained at 37 °C in a humified atmosphere with 5% CO_2_. In colony-forming tests, the majority of hDPSCs maintained their spindle-shaped morphology, consistent with other types of mesenchymal stem cells. All experimental procedures were performed under aseptic conditions.

### 2.2. Experimental Disks of Various Root Canal Sealers

The root canal sealers tested in this study were EndoSequence BC sealer (Brasseler), BioRoot RCS (Septodont), Endoseal MTA (Maruchi), and AH Plus (Dentsply DeTrey GmbH). Their compositions are from the manufacturer’s guidelines and are presented in Table 1. All experimental sealers were mixed in accordance with the manufacturer’s instructions. EndoSequence BC sealer and Endoseal MTA, a premixed sealer, were placed in a disposable syringe. One spoon of BioRoot RCS powder was mixed with five drops of a liquid consisting of water and calcium chloride. AH Plus Sealer was mixed at a 1:1 ratio in accordance with the manufacturer’s instructions. Disks of each root canal sealer 6 mm in diameter and 3 mm in height were made under aseptic conditions using sterile Teflon molds. All disks were stored in a 100% humidity chamber at 37 °C for 72 h to achieve setting. 

### 2.3. Cell Viability Assay 

**Figure 1:**
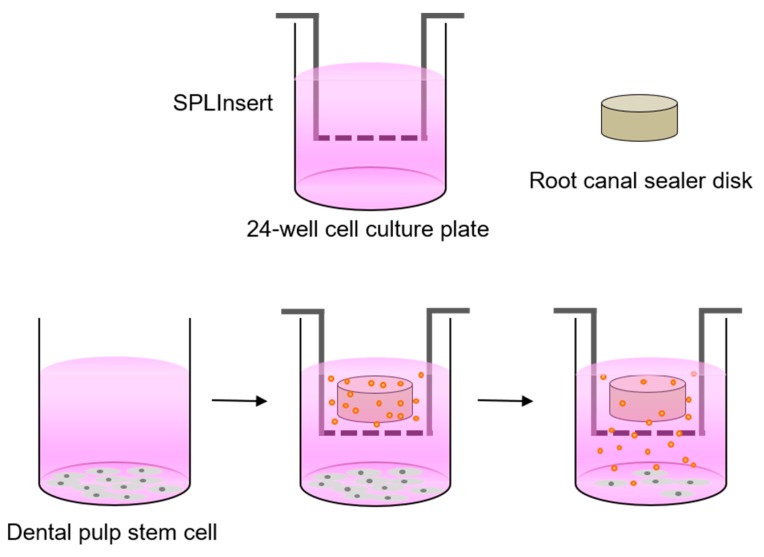
Experimental procedure of biocompatibility tests of each experimental sealer disk.

The cytotoxicity of various root canal sealers was evaluated using a methyl-thiazoldiphenyl-tetrazolium (MTT) assay (MTT Cell Growth Assay Kit, Chemicon, Rosemont, IL, USA). This assay exploits the ability of mitochondrial dehydrogenase enzymes to convert the yellow water-soluble tetrazolium salt 3-(4,5,-mimethyl-thiazol)-2,5,-diphenyl-tetrazolium bromide into colored compounds of formazan. The absorbance depends on the number of living cells. Here, hDPSCs were seeded in 24-well plates (SPL Life Sciences Co., Ltd., Pocheon, Korea) at a density of 1.0 × 10^4^ cells/well and incubated for 24 h in growth medium to allow for cell attachment. Each disk was placed individually into an insert with a 0.4-μm pore size (SPLInsert; SPL Life Sciences Co., Ltd.), and the insert was placed over the attached cells. To maintain the culture medium above the level of the disk, an additional 1 mL of growth medium was supplemented in each well. For controls, hDPSCs cultured without experimental disks were used. For 5 days, hDPSCs with various root canal sealer disks were incubated, and the growth medium was changed every 2 days. The proliferation of hDPSCs was analyzed after 0, 24, 48, 72, and 120 h of culture growth. Experimental procedures are shown in Figure 1. Wells were rinsed with phosphate-buffered saline (PBS) and incubated with the MTT solution (500 μg/mL) for 4 h. Subsequently, each well was rinsed with PBS, and dimethyl sulfoxide was then added to extract and solubilize the formazan. The absorbance at 570 nm was measured with an absorbance microplate reader (Power Wave XS; BioTek Instruments, Winooski, VT, USA) using absorbance at 630 nm as the reference wavelength. Each experimental group was analyzed in quadruplicate.

### 2.4. Cell Migration Assay

To evaluate cell migration ability, a scratch wound healing assay was used: hDPSCs were seeded in 24-well plates (SPL Life Sciences Co., Ltd.) at a density of 3.5 × 10^4^ cells/well and incubated for 24 h in growth medium to allow for cell attachment. A scratch was made in the center of the confluent layer of cells using a 1000-μL pipette tip. After wounding, cell debris was washed away with PBS. Each disk was placed individually into an insert with a 0.4-μm pore size (SPLInsert; SPL Life Sciences Co., Ltd.), and the insert was placed over the attached cells. To maintain the culture medium above the level of the disk, an additional 1 mL of growth medium was added to each well. For controls, hDPSCs cultured without experimental disks were used. For 4 days, hDPSCs with various root canal sealer disks were incubated, and the growth medium was changed every 2 days. Images of the scratch area were taken at 0, 24, 48, 72, and 96 h using a phase-contrast microscope (Olympus, Tokyo, Japan). ImageJ 1.46r software (National Institutes of Health, Bethesda, MD, USA) was used to measure the surface covered by the cells. The area of cell migration into the scratch area was calculated using the original scratch area as the reference. Each experimental group was analyzed in quadruplicate.

### 2.5. Cellular Morphology Evaluation

Here, hDPSC morphology was evaluated with a scanning electron microscope (SEM) after direct exposure of the cells to the experimental disks. Cells were seeded at a density of 5.0 × 10^4^ cells/well onto disks in 24-well plates (SPL Life Sciences Co., Ltd.), while control group cells were seeded on glass coverslips (SPL Life Sciences Co., Ltd.). After direct contact for 72 h, the growth medium was aspirated, and disks were washed using PBS. Cells on the disk were fixed by adding 2 mL of 2% buffered paraformaldehyde for 4 h and were then washed four times with PBS. All experimental disks with cells were rinsed with distilled water and dehydrated. Subsequently, they were mounted on aluminum stubs and sputter-coated with a 30-nm layer of gold. All specimens were observed with an S-4700 field emission SEM (FESEM; Hitachi, Tokyo, Japan). The voltage was set to 15.0 kV, the signal type was secondary electrons, the working distance was 12 mm, and the scan speed was 16 frames per 20 s. 

### 2.6. Alizarin Red Staining Assay

Each experimental root canal sealer disk was transferred to conical tubes containing 20 mL of fresh growth medium and stored in a humidified atmosphere at 37 °C and 5% CO_2_ for 7 days. Supernatant from this preparation was filtered using 0.20-μm filters (Minisart; Sartorius Stedim Biotech, Goetingen, Germany), and hDPSCs were seeded in 24-well plates (SPL Life Sciences Co., Ltd.) at a density of 2.0 × 10^4^ cells/well and incubated for 24 h in growth medium to allow for cell attachment. Mineralization activity was determined after 15 days of incubation, and root canal sealer extracts were changed every 3 days. Cells were stained with 2% Alizarin red stain solution for 20 min and washed five times with sterile water. The stain was treated with 10% cetylpyridinium chloride for 15 min to quantitatively evaluate the results, and the absorbance at 560 nm was measured with an absorbance microplate reader (Power Wave XS). Each experimental group was analyzed in quadruplicate.

### 2.7. Statistical Analysis

Statistical analysis was performed using SPSS software (ver. 24.0; IBM Corp., Armonk, NY, USA). The Shapiro–Wilk normality test was used to verify the data distribution. The data were normally distributed, and thus repeated measures analysis of variance was used for overall comparisons of cell viability and cell migration assays. Independent *t*-tests were used for pairwise comparisons of experimental groups in each experimental period. One-way analysis of variance and Tukey post hoc tests were used for the Alizarin red staining assay. *P-*values < 0.05 were considered significant.

## 3. Results

**Figure 2:**
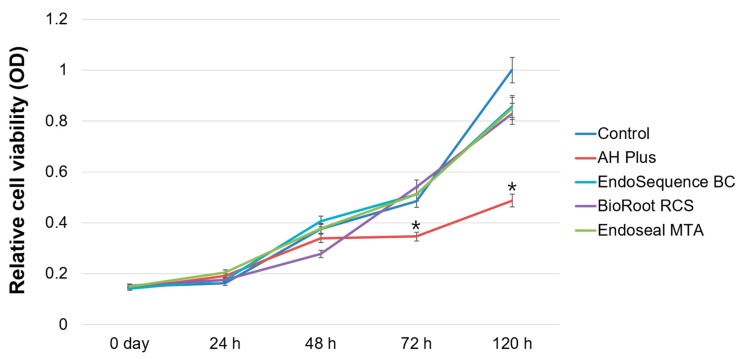
Relative cell viability rate based on a methyl-thiazoldiphenyl-tetrazolium (MTT) assay. Asterisks indicate statistically significant differences between the control group and experimental groups.

In the cell viability assay, there were no significant differences between the EndoSequence BC Sealer, BioRoot RCS, Endoseal MTA, and control groups in any experimental period (*p* > 0.05). Among all experimental groups, AH Plus showed the lowest cell viability after 72 h (*p* < 0.05) (Figure 2).

**Figure 3:**
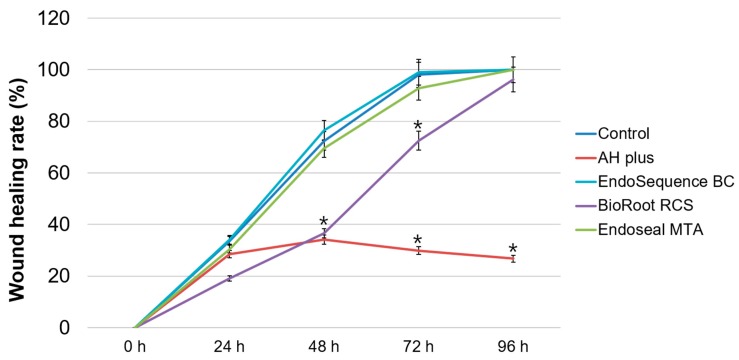
Relative wound healing rate based on the cell migration assay. Asterisks indicate statistically significant differences between the control group and experimental groups.

**Figure 4:**
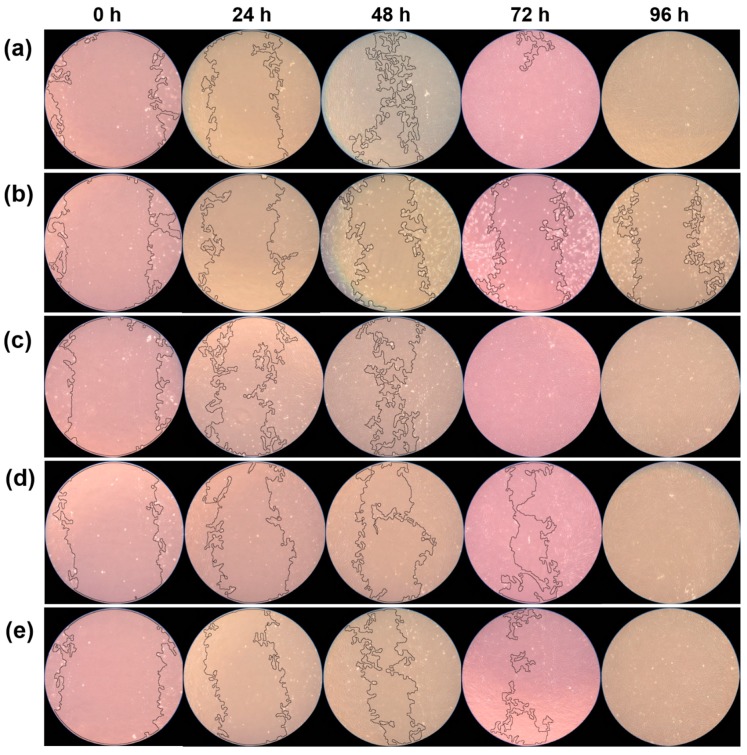
Representative image of wound healing percentage based on the cell migration assay. (**a**) Control group, (**b**) AH Plus group, (**c**) EndoSequence BC Sealer group, (**d**) BioRoot RCS group, (**e**) Endoseal MTA group.

In the cell migration assay, there were no significant differences between the EndoSequence BC Sealer, Endoseal MTA, and control groups in any experimental period (*p* > 0.05). BioRoot RCS exhibited slower cell migration than EndoSequence BC Sealer and Endoseal MTA did up to 72 h (*p* < 0.05). Conversely, it showed a similar wound healing percentage at 96 h (*p* > 0.05). Wound healing was not observed in the AH Plus group after 24 h, and there were significant differences between the AH Plus and control groups at 48 h, 72 h, and 96 h (*p* < 0.05) (Figure 3). Representative images of the wound healing rates of all experimental sealers, created using ImageJ 1.46r software, are shown in Figure 4.

**Figure 5:**
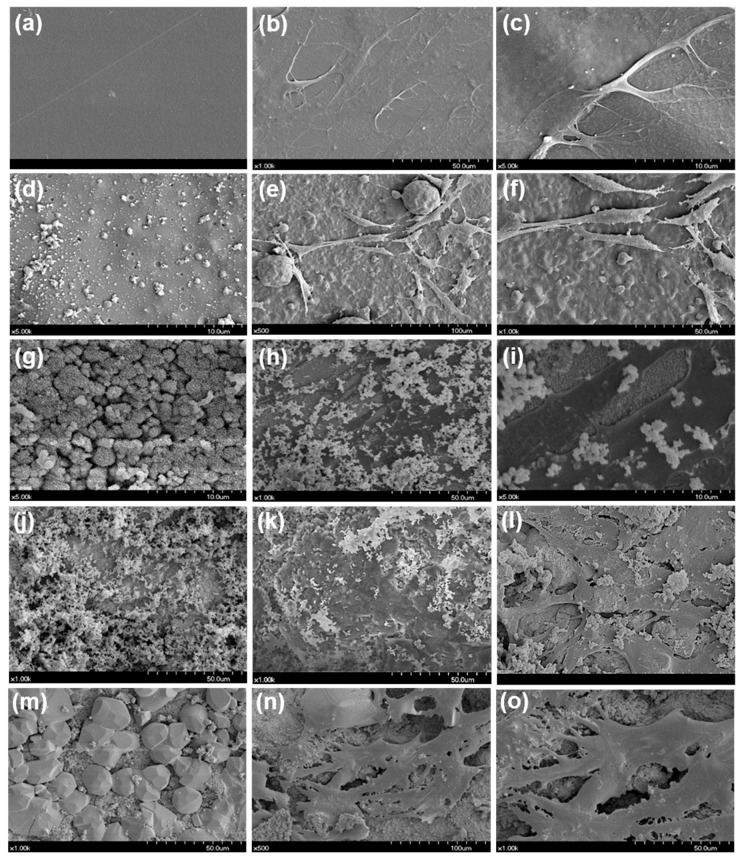
Scanning electron microscope (SEM) results of direct contact of human dental pulp stem cells (hDPSCs) with each experimental sealer. (**a**) Control group, (**b,c**) cells with control disk, (**d**) AH Plus disk, (**e,f**) cells with AH Plus disk, (**g**) EndoSequence BC Sealer disk, (**h,i**) cells with EndoSequence BC Sealer disk, (**j**) BioRoot RCS disk, (**k,l**) cells with BioRoot RCS disk, (**m**) Endoseal MTA disk, (**n,o**) cells with Endoseal MTA disk.

In the evaluation of cell morphology, hDPSCs in direct contact with EndoSequence BC Sealer, Endoseal MTA, and BioRoot RCS disks showed superior spreading compared to those in contact with the AH Plus disk. The EndoSequence BC Sealer and BioRoot RCS groups showed large amounts of small sealer particles on the disk surfaces but exhibited superior cell attachment compared to the AH Plus group (Figure 5).

**Figure 6:**
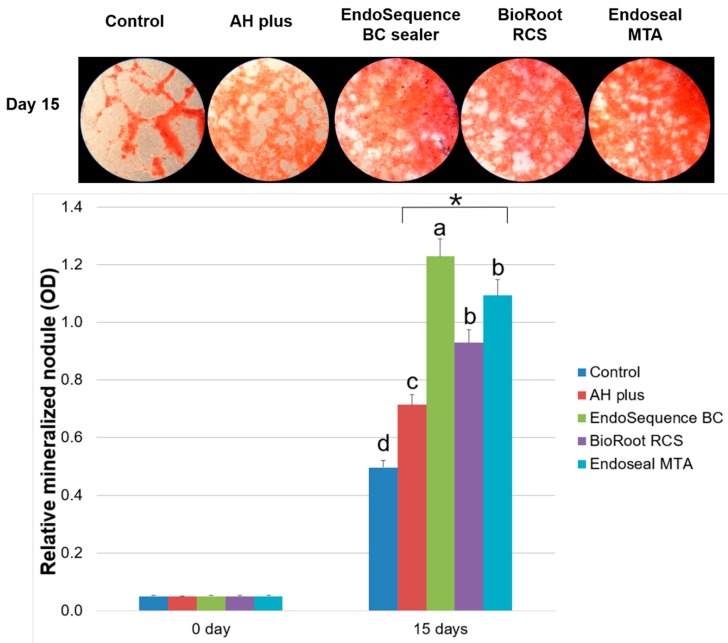
Relative mineralized nodule formation rate based on Alizarin red staining assay. Asterisk indicates a statistically significant difference between the control group and experimental groups. Different superscript letters indicate statistically significant differences.

In the Alizarin red staining assay, hDPSCs exposed to EndoSequence BC, BioRoot RCS, and Endoseal MTA extracts showed a significant increase in calcium nodule formation compared to the AH Plus group at 15 days (*p* < 0.05) (Figure 6).

## 4. Discussion

After proper nonsurgical root canal treatment, periapical wound healing occurs, with the removal of dead bacteria and necrotic tissue, formation of fibrovascular granulation tissue, and repair or regeneration of the injured area. During the healing process, osteoprogenitor and mesenchymal cells can undergo proliferation after stimulation by growth factors and cytokines, followed by differentiation into osteoblasts and production of bone matrix [24]. Biocompatible and bioactive root canal sealers have the potential to promote the rapid healing of apical periodontitis. The purpose of this study was to compare the biocompatibility and mineralization activity of three calcium silicate-based root canal sealers to those of a conventional resin-based sealer using human dental pulp stem cells.

In this study, the biocompatibility of EndoSequence BC Sealer, BioRoot RCS, Endoseal MTA, and AH Plus was analyzed using an MTT assay and a wound healing assay. In the present study, EndoSequence BC Sealer, BioRoot RCS, and Endoseal MTA showed superior cell viability and migration ability compared to AH Plus. EndoSequence BC Sealer is a novel bioceramic tricalcium silicate-based sealer that has shown nontoxic and biocompatible results in previous studies [10,13,17,25,26]. It is a premixed sealer that will not change in terms of its properties during the mixing procedure [17]. Compared to AH Plus, EndoSequence BC Sealer had less cytotoxicity in mouse fibroblasts [10] and in human bone marrow cells [17]. In three-dimensional cell culture models, EndoSequence BC Sealer showed the lowest cytotoxicity compared to AH Plus and MTA Fillapex (Angelus, Londrina, Brazil) [26]. BioRoot RCS is another novel bioceramic endodontic sealer, which has shown significantly lower cytotoxicity compared to other sealers, such as MTA Fillapex and SimpliSeal (Kerr, Orange, CA, USA) [27]. BioRoot RCS is composed of a powder consisting of tricalcium silicate and zirconium oxide and a liquid that is water-based and has calcium chloride and polymer additives. In an evaluation of direct contact with cells, BioRoot RCS was not cytotoxic and did not affect cell viability or morphology, which indicated that cell growth was not disturbed [18,19,28,29]. BioRoot RCS had fewer toxic effects on periodontal ligament cells [18] and A4 mouse pulp stem cells [19] than Pulp Canal Sealer did (CybronEndo, Orange, CA, USA). It also showed the lowest DNA double-strand breaks when compared to other resin- and silicate-based root canal sealers [28]. In previous studies, Endoseal MTA has shown satisfactory biocompatibility similar to that of ProRoot MTA [20,30]. In a histological evaluation, inflammatory scores of Endoseal MTA were also similar to those of ProRoot MTA, but were lower than those of AH Plus [20]. However, Collado-González et al. showed low rates of cell proliferation and viability at concentrations of 100% and 50% in an Endoseal MTA group [29]. In their study, SEM analysis showed restricted human periodontal ligament cell attachment on Endoseal MTA disks [29]. Endoseal MTA showed high levels of aluminum and contained bismuth, which are typical compositions of Portland cement [31], whereas BioRoot RCS did not show these elements because it is composed of pure tricalcium silicate [31]. Further studies are required to confirm the characterization of Endoseal MTA and BioRoot RCS. 

The morphology of cells in contact with EndoSequence BC Sealer, BioRoot RCS, and Endoseal MTA disks was flattened and cell attachment was better than in cells in contact with AH Plus in the present study (Figure 5). This result was consistent with previous studies [19,30]. Mouse pulp stem cells in BioRoot RCS extract showed superior spreading compared to cells in AH Plus extract [19]. In addition, MG-63 cells and human gingival fibroblasts cultured in direct contact with the AH Plus disk seemed to be less flattened and exhibited inferior spreading compared to those in the Endoseal MTA group [30]. AH Plus contains mutagenic substances in its composition, namely epoxy resin and an amine. The epoxy resin in AH Plus may cause breaks in the chain of cellular DNA. The higher cytotoxicity when it is freshly mixed is due to the initial release of minute amounts of formaldehyde and the epoxy resin component [7,10,32]. 

In this study, the mineralization activity of calcium silicate-based root canal sealers was evaluated using an Alizarin red staining assay. We found that not only calcium silicate-based root canal sealers but also AH Plus enhanced calcium nodule formation. However, hDPSCs exposed to EndoSequence BC, BioRoot RCS, and Endoseal MTA extracts showed a significant increase in calcium nodule formation compared to the AH Plus group (Figure 6). A previous study reported that alkaline pH may influence the biocompatibility and antibacterial ability of calcium silicate-based sealers [12]. It could also neutralize lactic acid from osteoclasts and prevent the dissolution of mineralized elements of teeth. In this regard, EndoSequence BC Sealer and BioRoot RCS could promote hard tissue formation [12,18,19]. Furthermore, extended Ca^2+^ release was observed when EndoSequence BC Sealer was used compared to AH Plus [33]. Calcium ions play an essential role in the development of hard tissue and mineralization activity. This extended Ca^2+^ release may explain why calcium silicate-based sealers are bioactive and affect the differentiation of bone marrow stem cells and osteoblast progenitor cells [34,35]. 

Some limitations have to be considered about the results of this study. First, we used dental pulp stem cells instead of bone morphogenic stem cells. Calcium silicate-based sealers were developed with the expectations of ideal cell proliferation and differentiation of apical tissue. Second, the experimental materials in this study were tested in a setting state, and a comparison between set and freshly mixed materials was not made. Third, physicochemical properties such as radiopacity, solubility, flowability, setting time, and dimensional stability were not evaluated in this study. 

All calcium silicate-based root canal sealers tested in this study showed good biological properties and mineralization activity compared to conventional resin-based sealer. However, EndoSequence BC Sealer had some disadvantages, such as an increased setting time and a requirement for moisture from dentinal tubules for complete setting [36]. In a previous study, EndoSequence BC Sealer required 72 h to achieve initial setting and 240 h to achieve final setting without water [37]. In addition, BioRoot RCS and Endoseal MTA showed lower cell viability at higher concentrations of sealer extracts [17,29]. Therefore, to confirm the beneficial use of calcium silicate-based sealers in root canal treatments, further in vitro and in vivo studies are required.

## 5. Conclusions

Calcium silicate-based root canal sealers such as EndoSequence BC Sealer, BioRoot RCS, and Endoseal MTA showed good cell viability and cell migration ability relative to the control group. The AH Plus group showed the lowest cell viability and no wound healing. All experimental calcium silicate-based sealers exhibited increased mineralization activity compared to AH Plus.

## Figures and Tables

**Table 1:** Manufacturer and chemical composition of experimental sealers.

Table	Manufacturer	Composition	Batch Number
AH Plus	Dentsply DeTrey GmbH, Konstanz, Germany	Epoxide paste: diepoxide, calcium tungstate, zirconium oxide, aerosil, pigment; amine paste: 1-adamantane amine*, N, N’-*dibenzyl-5-oxa-nonandiamin-1,9, TCD-diamine, calcium tungstate, zirconium oxide, aerosil, and silicon oil	1703000226
EndoSequence BC Sealer	Brasseler, Savannah, GA, USA	Zirconium oxide, calcium silicates, calcium phosphate monobasic, calcium hydroxide, filler and thickening agents	17004SP
BioRoot RCS	Septodont, Saint Maur-des-Fossés, France	Tricalcium silicate, zirconium oxide (opacifier) and excipients in its powder form, and calcium chloride and excipients as an aqueous liquid	B16422
Endoseal MTA	Maruchi, Wonju, Korea	Calcium silicates, calcium aluminates, calcium aluminoferrite, calcium sulfates, radiopacifier, and thickening agents	CD180327D
